# Beyond Spelling: Oral and Written Expository Discourse Skills in Adolescents With Dyslexia

**DOI:** 10.1002/dys.70036

**Published:** 2026-06-14

**Authors:** Helena Oliv, Anna Eva Hallin

**Affiliations:** ^1^ Department of Clinical Science, Intervention and Technology (CLINTEC), Division of Speech and Language Pathology Karolinska Institutet Stockholm Sweden; ^2^ Department of Clinical Sciences Karolinska Institutet Danderyd Hospital Stockholm Sweden

**Keywords:** adolescents, dyslexia, expository discourse, modality differences, spelling, writing

## Abstract

Students with dyslexia may produce shorter written texts with poorer content and less complex language than peers, but it remains unclear whether such differences reflect increased writing effort associated with dyslexia or co‐occurring non‐phonological language difficulties. Therefore, this study compared oral and written discourse skills in Swedish adolescents (11–16 years old) with dyslexia (*n* = 16) and typical development (*n* = 37). Each participant explained a sport or game of their choice—first orally and, on a later occasion, in writing. Samples were analysed for productivity, syntactic complexity, linguistic and spelling accuracy and content. No significant group differences were found in either task apart from spelling. Across groups oral samples had higher linguistic accuracy, more words and more content than written samples. The results indicate that the spelling and decoding abilities that characterise dyslexia are not associated with poorer content, productivity, complexity or accuracy of written or oral samples and highlight the importance of a thorough oral language evaluation when students present with written language difficulties beyond spelling. Future studies should compare discourse skills in adolescents with dyslexia only, dyslexia with co‐occurring developmental language disorder/DLD and DLD only, to further investigate the role of non‐phonological language skills in oral and written discourse production.

## Practitioner Points

- Our sample of adolescents with dyslexia performed comparably to peers in an oral and written expository task, apart from spelling
- Both groups produced oral samples with more words, higher linguistic accuracy and more content than their written samples.
- In a written task with sufficient support, dyslexia itself does not seem to cause difficulties with productivity, content, or grammar.
- Writing difficulties beyond spelling in adolescents with dyslexia may indicate co‐occurring language weaknesses and warrant comprehensive oral language assessment
- Future studies should compare adolescents with dyslexia, DLD and co‐occurring conditions to clarify non‐phonological influences on oral and written discourse production.

## 1 Introduction

Oral and written discourse skills are crucial for social participation and academic performance. Students are expected to demonstrate their knowledge in all subjects through reasoning in either oral or written form. These demands pose particular challenges for students with language, reading and writing difficulties, for example, dyslexia. Dyslexia is characterised by deficits in written language, specifically word decoding and spelling (Catts et al. 2024). However, evidence suggests children and adolescents with reading difficulties (word reading and/or reading comprehension difficulties) also show significantly lower performance on several measures of writing compared to both age‐matched and reading‐matched peers (Graham et al. 2021) and that dyslexia may be associated with broader language vulnerabilities in both the written and oral modality (Adlof and Hogan 2018). Previous research on individuals with dyslexia has focused more on the written modality, however and more on reading than writing problems, even though writing difficulties are common and often persist into adulthood (Berninger et al. 2008; Connelly et al. 2006). Writing places high demands on linguistic, cognitive and executive resources and weaknesses in these skills might consequently affect written text production, beyond difficulties with spelling (Berninger and Amtmann 2003).

Spoken and written language samples are useful tools for educators and speech and language pathologists (SLPs) to assess adolescents' use of language during activities that are relevant to the academic curriculum (Heilmann et al. 2020; Lundine 2020; Price and Jackson 2015). In the Swedish clinical guidelines for assessment of reading and writing difficulties (Slof 2017), assessment of oral and written discourse skills is recommended as part of a comprehensive language, reading and writing evaluation. However, there is a lack of standardised Swedish discourse tasks suitable for adolescents, and research is limited regarding how adolescents with dyslexia perform in oral and written discourse tasks compared to peers with typical development (TD).

In the present study, oral and written expository discourse skills are investigated in Swedish students in grades 6–9 with and without dyslexia. The expository genre was selected because it is commonly used by adolescents in everyday interaction and taught and assessed in school (Lundine and McCauley 2016; Lundine 2020). In addition, expository tasks (e.g., description, explanation, comparison or persuasion) have been shown to elicit more complex language from adolescents in comparison with both narrative tasks and conversational tasks (Nippold et al. 2005, 2008; Scott and Windsor 2000). The use of complex syntax can increase the efficiency of communication and enables a person to express a message in an organised way (Scott and Balthazar 2010). For this reason, expository tasks are well suited for evaluating more complex language abilities and including an expository task in evaluations can guide what support is needed for students to show their knowledge in school in, for example, oral presentations and written exams.

A well‐researched expository task is the ‘Favourite game or sport’ (FGS) task, in which participants describe how to play their favourite game or sport (Nippold et al. 2005). Children may produce more complex language when they have background knowledge of and personal interest in a subject (Nippold 2009; Lundine and McCauley 2016) and describing a favourite sport or game is considered to carry relatively low cultural bias since sports or games exist in all cultures (Heilmann and Malone 2014). The FGS task has been used in several studies to elicit oral expository discourse in younger and older English‐speaking school‐age children (Heilmann and Malone 2014; Nippold et al. 2008; Westerveld and Moran 2013) and a Swedish translation and adaptation of the task was used in the present study.

Language samples can be analysed at both micro‐ and macrostructural levels. Microstructure refers to the morphosyntactic and semantic characteristics of a sample and the measures include, for example, productivity, syntactic complexity and linguistic errors. Macrostructure refers to the content and organization of a language sample. The microstructural and macrostructural abilities required to produce expository discourse are gradually developing throughout the school years (Kay‐Raining Bird et al. 2016; Lundine and McCauley 2016; Nippold et al. 2005). Macrostructure measures have been well studied in narrative production but have received limited attention in the analysis of expository discourse (Guilkey and Wagovich 2022).

### 1.1 Oral and Written Language Skills in Adolescents With and Without Dyslexia

Dyslexia is primarily defined by persistent decoding and spelling difficulties, with relative strengths and weaknesses depending on orthography (Moore et al. 2023). The difficulties are often associated with deficits in phonological processing, including poor phonological awareness, limited verbal short‐term memory and/or problems with rapid automatized naming (Ramus et al. 2013). In Swedish, a language with a semi‐transparent alphabetic orthography, both decoding (accuracy and speed) and spelling are typically negatively affected in individuals with dyslexia. Orthographic challenges in Swedish include prevalent consonant clusters, long compound words and several consonant phonemes associated with irregular and varied spelling patterns (Svensson et al. 2024). As mentioned previously, research has indicated that individuals with dyslexia may also struggle with other aspects of language and executive functioning (Adlof and Hogan 2018; Bishop et al. 2009; Lonergan et al. 2019; Ramus et al. 2013), although a recent systematic review highlighted that studies frequently fail to adequately assess or report oral language abilities in their participants with dyslexia, making it difficult to determine whether reported broader language weaknesses reflect dyslexia itself or co‐occurring language difficulties (Cabbage et al. 2026). Comorbidity between dyslexia and developmental language disorder (DLD) is also very common, with estimates ranging from 17% to 71% (Adlof and Hogan 2018). DLD involves unexpected, significant and pervasive language difficulties in several phonological and non‐phonological language domains despite adequate language exposure and cognitive abilities (Bishop et al. 2017). Importantly, DLD is not characterized by persistent decoding difficulties. Ramus et al. (2013) showed that students with comorbid dyslexia and DLD showed marked difficulties in both non‐phonological language skills and phonological skills/phonological representations, whereas students in a dyslexia only group showed non‐phonological language skills below but within 1 SD of the TD group mean. The same group exhibited phonological skills that were much lower than those of the TD group.

Thus, evidence suggests that even in the absence of co‐occurring DLD, children with dyslexia may show subtle but measurable weaknesses in non‐phonological language skills. Bishop et al. (2009) found that children with dyslexia who did not meet criteria for DLD still showed significantly poorer vocabulary, sentence repetition and syntactic comprehension scores than TD children, although their standardised scores in these tasks were within normal limits on average and their oral narrative skills were comparable to TD children. Altmann et al. (2008) reported difficulties in late‐developing syntactic skills among children with dyslexia and morphological problems in children with dyslexia have also been described (Rispens and Been 2007; Robertson et al. 2012). All these authors discuss the importance of phonological processing skills for syntactic, morphological and lexical development and that this might be the cause of the non‐phonological linguistic weaknesses seen in individuals with dyslexia. Consequently, these linguistic weaknesses might lead to reduced complexity and accuracy in more advanced oral and written discourse tasks, although, to our knowledge, only written expository skills, not oral, have been explored in adolescents with dyslexia previously.

A theoretical starting point in understanding the potential challenges in written discourse in students with dyslexia can be found in the model ‘The simple view of writing’ (Berninger and Amtmann 2003). The model highlights transcription (spelling, handwriting/keyboarding), executive functions (planning, organization, self‐regulation) and text generation as three key components of writing, all supported by working memory. When transcription skills are sufficiently automatized, more cognitive resources are available for higher‐level composing skills like planning and revising. For students with dyslexia, the combination of transcription and decoding difficulties, together with potentially weakened non‐phonological language skills and executive functions, may affect written language negatively. Problems in transcription skills might lead to more pauses and slower writing and consequently shorter texts (Connelly et al. 2006; Sumner et al. 2013). Deficits in working memory and executive functioning may further negatively affect text organization and coherence (Berninger et al. 2008; Herbert et al. 2018). In addition, impaired decoding skills may hinder effective revision (Hayes 1996; Herbert et al. 2018).

Previous studies directly examining written discourse skills in adolescents with dyslexia have yielded mixed results, however. In studies analysing handwritten samples, Sumner and Connelly (2020) observed that university students with dyslexia showed no differences in measures of time spent on writing and amount of text produced compared to their peers when given an expository essay prompt, but did show weaker punctuation, grammar and sentence structure. They also showed difficulties on macrolevel measures, with weaker organization and coherence, although this conflicts with an earlier study including university students with dyslexia (Connelly et al. 2006). Puranik et al. (2007) examined the writing performance of adolescents with dyslexia, DLD and TD (11–21 years old) in an expository retelling task. Participants listened twice to a social science text and then wrote down what they remembered. In this task, the adolescents with dyslexia produced more spelling and grammatical errors than their typically developing peers on average, but did not differ in syntactic complexity or text length. Finally, in a Swedish study where students typed a persuasive expository text in response to a short film depicting various problems from a school day, adolescents with reading and writing difficulties produced shorter texts with lower text quality and more misspelled words than TD peers (Wengelin et al. 2014). Similar to Puranik et al. (2007), there were no significant group differences in terms of syntactic complexity in this study.

Modality‐related differences have been investigated in children and adolescents with TD (Brimo and Hall‐Mills 2019; Fey et al. 2004; Scott and Windsor 2000). Scott and Windsor (2000), found that written summaries of young school children with TD were shorter, produced at a slower rate and had more grammatical errors than spoken versions, but no modality differences were found in the production of complex syntax. In contrast, Brimo and Hall‐Mills (2019) found that adolescents with TD produced a higher percentage of complex utterances in written expository samples compared to spoken expository samples. The researchers suggested that differences may be dependent on genre, age and reading and writing proficiency. Consistent with this suggestion, Fey et al. (2004) reported that children in the early school years typically produce oral narratives that are longer, more complex and more grammatically correct than their written narratives. They further observed that the greatest improvement in the written modality in terms of text length, complexity and general quality occurred between second and fourth grade, as a result of the reading and writing instruction during the early school years. Again, to our knowledge, there are no similar studies comparing oral and written modalities in dyslexia.

To summarise, there is limited research on written expository skills in adolescents and comparisons across studies are complicated by methodological differences, including variation in the tasks used, analyses and whether comorbid language difficulties are controlled for (Cabbage et al. 2026). Existing research suggests that texts produced by students with dyslexia typically contain a higher proportion of spelling errors than those of peers. Most studies report no group differences in syntactic complexity in written samples, but others report weaknesses in grammatical accuracy, text length and organization. However, it remains unclear whether these difficulties reflect transcription and decoding demands which are higher for students with dyslexia or co‐occurring non‐phonological language difficulties. This issue can be clarified by comparing oral and written discourse skills using the same matched tasks in students with dyslexia while controlling for co‐occurring language difficulties. This knowledge is important for clinicians seeking a comprehensive picture of the non‐phonological language abilities of students with dyslexia and consequently what support these students might need in academic settings apart from reading and spelling.

## 2 Aims, Research Questions and Hypotheses

Thus, the aims of the present study were to compare and describe oral and written expository discourse in Swedish adolescents with and without dyslexia and to investigate the effect of modality (oral/written) on both microlevel and macrolevel measures in both groups. The study was guided by the following research questions:

1. What similarities and differences are there in total number of words, syntactic complexity, linguistic errors, and content, as well as spelling errors in the written sample, between adolescents with dyslexia and age‐matched peers with typical development (TD) in matched oral and written expository tasks?
2. How does modality (oral/written) affect total number of words, syntactic complexity, linguistic errors and content, and does the effect differ between the two groups (dyslexia/TD)?

Given that expository discourse is a demanding linguistic task, adolescents with dyslexia may show weaknesses in both tasks relative to peers due to subtle non‐phonological language difficulties. Further, adolescents with dyslexia may exhibit a pattern similar to younger peers (cf. Fey et al. 2004), where oral samples are longer, more syntactically complex and have higher linguistic accuracy than written samples, which would indicate that transcription and decoding demands are affecting writing quality beyond spelling. This pattern may be less pronounced in the TD group due to more developed writing skills.

## 3 Methods

This study is part of the larger project EXPLORE‐SA: Expository language and oral retelling in Swedish adolescents (PI: AE. Hallin), approved by the Stockholm Regional Ethical Review board (#2017/49‐31/4). Caregivers signed a consent form prior to participation. Students received both written and oral information and were asked for oral consent before testing began.

### 3.1 Participants

Fifty‐three participants were recruited for this study, 16 with dyslexia (10 girls and 6 boys) and 37 with typical development (TD) (26 girls and 11 boys). All participants in the TD group and two of the participants in the dyslexia group were recruited through principals and teachers in schools in the larger metropolitan area of Stockholm. An additional 14 students with dyslexia were recruited via a large speech and language clinic in Stockholm. Participants with previously diagnosed difficulties (as reported by caregivers) such as developmental language disorder (DLD), autism, ADHD and intellectual disability were excluded. All participants were students in Swedish grades 6–9 (11;10–16;2 years old) and were native speakers of Swedish, that is, they had been exposed to Swedish from birth, were born in Sweden and had at least one Swedish‐speaking parent. All students in the dyslexia group and all but three participants in the TD group were only exposed to Swedish at home. Three participants in the TD group were exposed to one additional language at home (simultaneous bilinguals). Participants included in the TD group had typical general language, reading and writing development as reported by caregivers in a background questionnaire. Participants included in the dyslexia group had received a dyslexia diagnosis following a comprehensive language, reading and writing evaluation by an SLP, where a comorbid diagnosis of developmental language disorder (DLD) had been ruled out. Although we do not have specific information on whether the participants with dyslexia had participated in any reading interventions or not prior to the evaluation, these interventions would have been focused on decoding, since individual or small group language intervention for school‐age children is extremely sparse in Sweden (NPO 2025). If previous intervention had resulted in significant gains, the students would not have qualified for a dyslexia diagnosis.

To enable a comparison between groups on selected standardised measures of language and working memory, all participants were assessed with TROG‐2 (Test for Reception of Grammar 2 (Bishop 2003), Swedish version; Holmberg and Lundälv 2002), Recalling sentences and Digit repetition from CELF‐4 (Semel et al. 2003) and Nonword repetition (Selin and Törnkvist 2006). Since the Swedish version of CELF‐4 does not have norms for children above 12:11, raw scores are reported. Results from independent two‐tailed *t*‐tests showed significant group differences on Recalling sentences (with a mean difference of raw scores corresponding to *d* = 0.89), which is in line with group differences reported in Bishop et al. (2009) and Digit repetition (*d* = 1.41). On TROG‐2 and the Nonword repetition task, group differences were not significant, see Table 1.

**TABLE 1:** Descriptive data for the participants included in the study.

Measure	Dyslexia (*n* = 16)		TD (*n* = 37)		*p* *‐value
	Mean (SD)	Range	Mean (SD)	Range	
Age (months)	169.75 (15.60)	146–194	165.32 (14.05)	142–188	0.313
TROG 2, Swedish version (percentile)	48.38 (16.80)	23–73	45.51 (19.22)	12–82	0.608
CELF‐4 Recalling sentences (raw score, max 54)	**36.94** (6.22)	24–50	**42.89** (7.17)	25–52	0.006
CELF‐4 Digit repetition (raw score, max 30)	**11.69** (2.89)	9–20	**13.66** (2.67)^a^	10–20	0.021
Nonword repetition (max 100 consonants correct)	94.50 (5.02)	81–100	95.89 (3.28)^a^	87–100	0.244

*Note:* Significant group differences are bolded.

* Groups compared with an independent, two‐tailed *t*‐test.

^a^ *n* = 35.

### 3.2 The Expository Tasks

The participants completed two expository tasks, one oral and one written, based on the Favourite Game or Sport (FGS) task, originally developed by Nippold et al. (2005). The elicitation instructions for the oral task are from Miller et al. (2016), translated by the second author, and those for the written task were adapted from the oral version.

### 3.3 Procedure

Data was collected either by an SLP master's student (first author) or a trained collaborator on the research project. The testing was performed on two occasions, either in a quiet room in the student's school or in an SLP clinic. On the first occasion, participants who were recruited from schools first completed a narrative task (not reported here), then standardised testing, then a second narrative task and finally the oral expository task. The whole session took 45–60 min. For students with dyslexia recruited from the SLP clinic, the standardised testing was administered in conjunction with their reading and writing evaluation and these test results were used in the study after informed consent. The oral expository task was completed towards the end of the second session of the reading and writing evaluation. The oral expository task took approximately 15 min to complete. During the task, the participant was asked to explain his or her favourite game or favourite sport to the examiner. The student was asked to imagine that the test leader was a child of the same age and who did not know the game/sport. The student would then plan his or her explanation by writing down key words including eight topics (object of contest, preparation, start, course of play, rules, scoring, duration and strategy) on a provided planning sheet. If needed, the examiner read the topics with descriptions aloud. With the planning sheet and their own notes as support, the students were then asked to orally explain their chosen sport or game with the instruction that they should speak for at least 5 min. If the student was done before 5 min had passed, the test leader asked: “Is there anything more you can tell me?” The explanation was recorded with a portable digital recorder (Tascam DR‐07MK2).

On a separate second occasion, the written expository sample was elicited individually or in groups of maximum five students, in a quiet room in the school or in the SLP clinic. The participants had their planning sheet from the oral expository task in front of them and were asked to write down an explanation of how to play the sport or the game and that they had 20 min to do so. The text was written by hand, similar to most examination situations in Swedish primary and secondary schools (digital examinations were not yet implemented at the time of data collection). After 20 min, students had an extra 5 min to finish if needed.

Due to a technical error, the oral sample is missing from one participant in the TD group. Written samples are missing from three participants in the TD group because they were absent from the second session.

### 3.4 Transcriptions and Analyses

The recordings were orthographically transcribed and coded for microlevel measures according to the Swedish addendum to the SALT manual (Hallin et al. 2019) for the Systematic Analysis of Language Transcripts software (SALT: Miller and Iglesias 2019). The Swedish addendum contained expanded explanations on how to judge and code linguistic errors in Swedish spoken language samples. Errors coded included word order errors, other syntactic errors such as omissions, morphological errors, pronoun errors and other word‐level semantic errors. Errors that are acceptable in spoken language, for example, certain word order errors after hesitation/maze and self‐corrected errors were not coded as errors. A total of five trained collaborators on the project (SLP students/master's students) transcribed and coded different oral samples from the participants with TD. The first author transcribed and coded all oral and written samples from participants with dyslexia, a majority of the oral samples from participants with TD, as well as all written language samples. Brief instructions for the written coding manual were further developed in this project by the first author, including specifications regarding written linguistic errors, spelling and punctuation errors and guidelines for how these should be coded including examples of utterances. Errors regarding punctuation and capitalization were not analysed or reported in this study. All transcripts, oral and written, were divided into *T‐units*, which is defined as a main clause with all its subordinate clauses (Hunt 1970). The measures included were (1) total number of words; (2) Mean length of T‐unit (MLTU); (3) Subordination of Index (SI: average proportion of complete clauses per T‐unit); (4) Percentage of T‐units with linguistic errors; and for the written samples (5) Proportion of misspelled words (the number of misspelled words in a student's written sample divided by total number of words).

All transcriptions were scored regarding content/structure (macrolevel measures) using the Expository Scoring Scheme (ESS) rubric (Miller et al. 2016), with a Swedish version developed within this project. With guidance from the ESS rubric, 10 components were rated using a 0–5 scale: The first eight components corresponded to the topics listed on the planning sheet that was given to students: *object*, *preparation*, *start*, *course of play*, *rules*, *scoring*, *duration* and *strategy*. In addition, the rater completed two additional global ratings of *terminology* (i.e., that terms of game/sport are used and that new terms are defined when used) and *cohesion* (i.e., overall flow of the sample, including order, covering topics completely and smooth transitions). For each component, 5 points were given for proficient use, 3 points for emerging or some use and 1 point for poor performance. A score of 0 was given for poor performance together with a variety of errors (e. g. explaining a different game or sport, not completing the task, or many abandoned utterances). Additional instructions of how to apply the ESS rubric were developed by the authors to ensure acceptable inter‐rater reliability. A total ESS score was calculated based on the sum of the ratings for the 10 components, with a maximum score of 50.

### 3.5 Interrater Reliability

For microlevel measures in the oral samples, a total of nine samples (17%) were randomly selected and re‐transcribed and re‐coded by different collaborators (SLP students and master's students) in the larger project, where each audio file was transcribed and coded by two different persons. This procedure was chosen to get a measure of reliability across all the different transcribers. Word‐for‐word agreement in the oral samples was 93%–100%. From all transcriptions, values for the variables in SALT were obtained and percentage agreement was calculated for each transcription. For microlevel measures in the written samples, six samples (12%) were randomly selected, re‐transcribed and re‐coded by the second author with guidance from the written sample coding scheme. For mean agreement values, see Table 2.

**TABLE 2:** Mean percentage agreement for the microlevel measures in reliability transcriptions of the oral and written tasks.

Task	Total number of words	Mean length of T‐units	Subordinate index	Proportion T‐units with errors	Proportion spelling errors
Oral FGS (mean agreement in percent, *n* = 9 samples)	95	94	94	71	—
Written FGS (mean agreement in percent, *n* = 6 samples)	99	97	95	85	91

Based on all reliability transcriptions (*n* = 15), the reliability for both total number of words and grammatical complexity of the oral and written samples were good (94% or higher). Proportion of T‐units with linguistic errors showed acceptable reliability for the written samples, but below the acceptable level for the oral task (which according to SALT is 85%).

For macrolevel analyses, the interrater reliability was calculated for the total ESS score. Five samples, two oral samples and three written samples (approximately 5% of the total number of samples included in this study) were randomly selected and re‐rated by the second author. The modified version of the ESS rubric (described above) was followed. Agreement between the first author and the second author was calculated as the mean percentage of agreement of the total ESS score for each sample and those percentages were then averaged. Interrater agreement for the total ESS score reached 91%.

### 3.6 Statistical Analyses

Statistical analyses were conducted using SPSS. Two‐way repeated measures ANOVAs were conducted to examine the effects of group and modality on all measures. Analyses were conducted on total number of words, syntactic complexity (MLTU and SI), proportion of T‐units with linguistic errors and content/structure (ESS rubric scores). Before analyses, assumptions for ANOVA were controlled and met. In the written sample, spelling was compared with an independent, two‐tailed *t*‐test. In addition, to further investigate both individual and group differences, the difference between the oral and written task was calculated for each measure and participant and averaged by group. The 95% confidence intervals were calculated and marked in figures where individual difference scores were also plotted to provide a visual indication of the variation within the sample as well as the magnitude of the overlap between the estimated means of the two groups.

## 4 Results

Table 3 presents an overview of the descriptive data for all measures, divided by group and task. Some variables showed a large range of scores within the groups. In the dyslexia group, the range in total number of words was greatest in the oral task, where the difference between the mean and median suggested a slight skew in the distribution. In particular, two participants with dyslexia produced notably lengthy oral explanations and showed large differences between the oral and written tasks in total number of words. For the measures of syntactic complexity, the mean and median differed only slightly between the groups, but the range in MLTU was greater in the TD group in both the oral and written tasks, where some participants produced T‐units with many words. The largest range in linguistic errors was found in the TD group across both tasks.

**TABLE 3:** Descriptive data from the oral and written task for the two groups.

Measure	Task	Dyslexia (*n* = 16)			TD (*n* = 37)^a^, ^b^		
		Mean (SD)	*Mdn*	Range	Mean (SD)	*Mdn*	Range
Total number of words	O	608.7 (228.3)	584	230–1121	565.4 (161.3)	579	155–913
	W	228.1 (62.5)	240	148–361	235.0 (80.7)	235	110–384
Mean length of T‐unit (MLTU)	O	11.4 (1.1)	11.6	9.3–13.2	11.9 (2.0)	11.5	8.0–17.7
	W	11.1 (1.5)	10.6	9.3–13.3	12.2 (2.9)	11.8	8.0–21.2
Subordination Index (SI)	O	1.7 (0.2)	1.7	1.2–2.0	1.7 (0.2)	1.7	1.3–2.2
	W	1.7 (0.2)	1.7	1.1–2.0	1.8 (0.3)	1.7	1.3–2.4
Percentage of T‐units w. errors	O	9.8 (6.9)	7.9	0–25.0	8.9 (7.6)	7.3	0–35.1
	W	16.2 (7.8)	16.2	0–31.3	12.2 (10.7)	10.6	0–50.0
Proportion of misspelled words	W	8.5 (11.5)	7.5	0.8–16.5	1.3 (1.5)	0.8	0–5.6
ESS score total (max 50)	O	32.0 (7.3)	34.0	16–44	32.7 (6.9)	32.0	18–48
	W	24.6 (5.7)	24.0	16–38	27.2 (7.0)	28.0	10–38

*Note:* O = Oral: W = Written.

^a^ *n* ^oral^ = 36.

^b^ *n* ^written^ = 34.

### 4.1 Productivity

#### 4.1.1 Total Number of Words

There was a significant main effect for modality, *F*(1, 47) = 195.08, *p* < 0.001, Wilks' Lambda (Λ) = 0.19, partial eta squared (ηp^2^) = 0.81, with participants producing significantly more words in the oral task (*M* = 578.8, SD = 183.3) than in the written task (*M* = 232.8, SD = 74.7). The main effect of group was not significant, *F*(1, 47) = 0.089, *p* = 0.77 and there was no significant interaction between group and modality, *F*(1, 47) = 0.72, *p* = 0.40. This suggests that modality affected the total number of words in this sample, but that there were no significant differences between the two groups.

**FIGURE 1:**
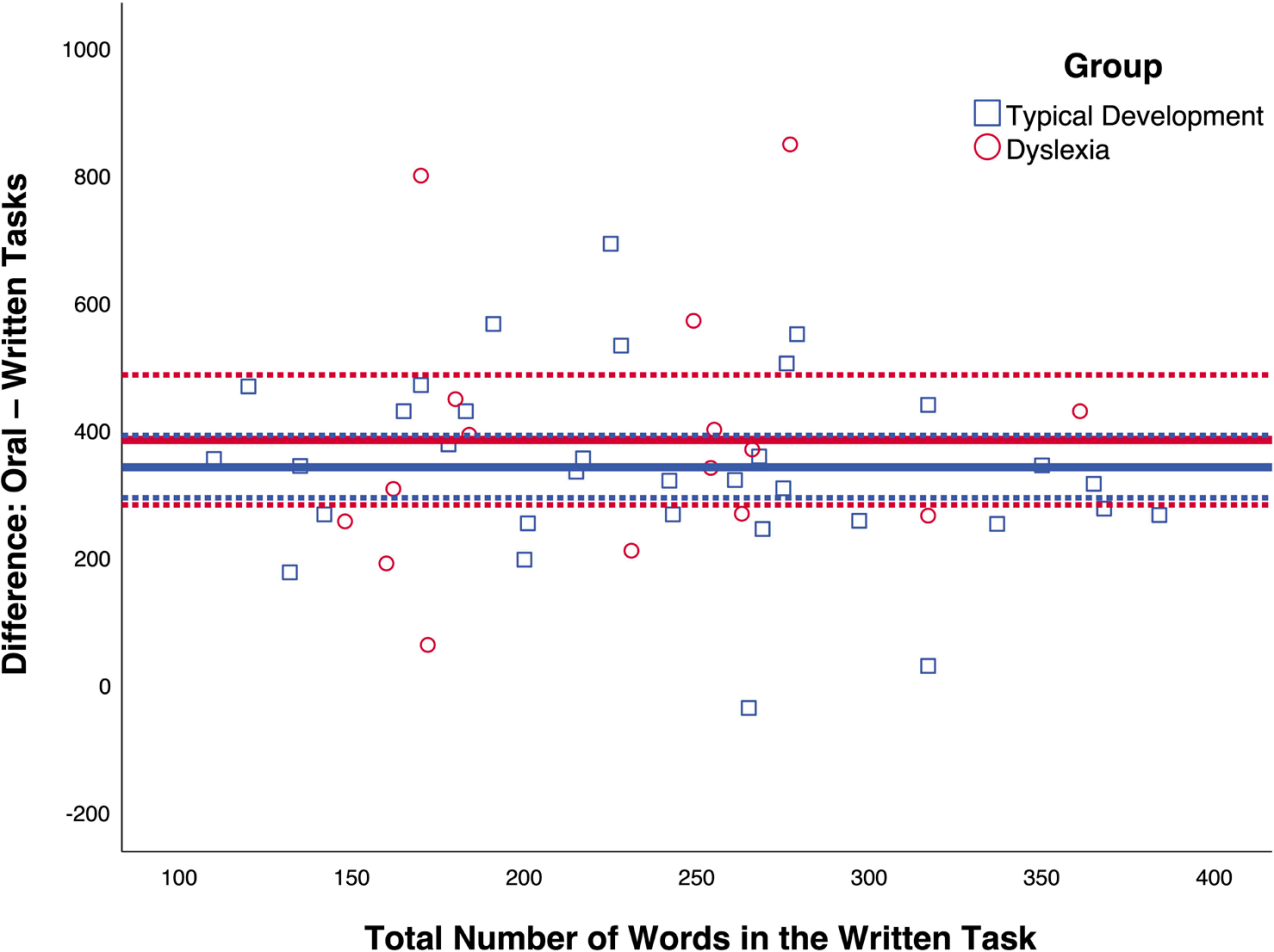
Difference between the oral and written tasks in terms of total number of words. Dots represent individual participants. The red solid line indicates mean difference for the dyslexia group (*M* = 380) and the blue solid line indicates mean difference for the TD group (*M* = 337). Dashed lines indicate upper and lower confidence intervals.

Figure 1 shows individual differences in the total number of words between the oral and written tasks for participants with results from both tasks in the dyslexia group (*n* = 16, mean difference = 380) and the TD group (*n* = 33, mean difference = 337). The 95% confidence intervals overlap completely, indicating that the difference is not statistically significant.

### 4.2 Syntactic Complexity

#### 4.2.1 Mean Length of T‐Unit (MLTU)

There was no significant main effect of modality *F*(1, 47) = 0.48, *p* = 0.827, no significant main effect of group *F*(1, 47) = 1.723, *p* = 0.196 and no significant interaction between group and modality *F*(1, 47) = 0.313, *p* = 0.579. This suggests that modality did not affect the average MLTU of the participants in this sample.

**FIGURE 2:**
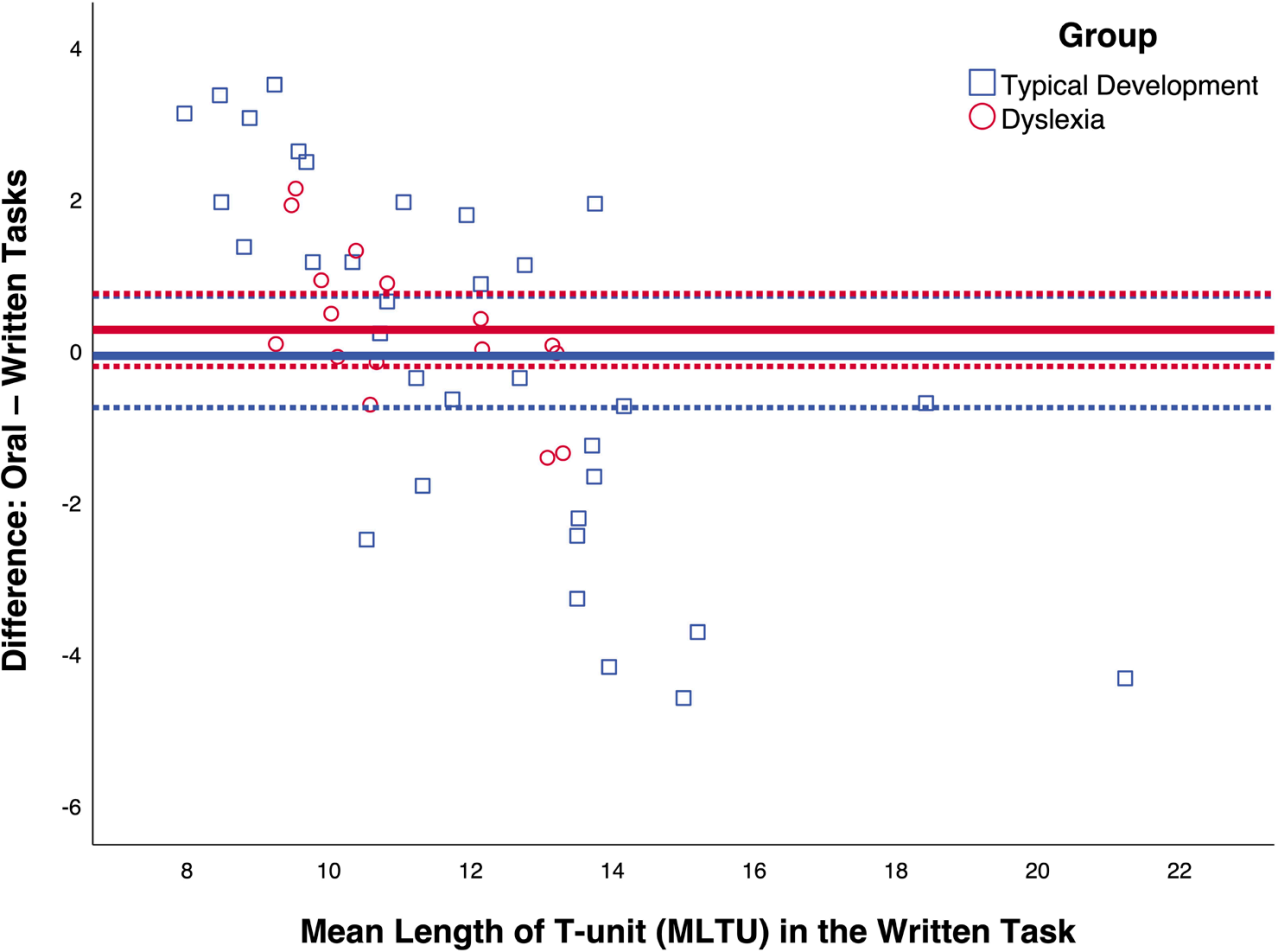
Difference between the oral and written tasks in terms of mean length of T‐unit (MLTU). Dots represent individual participants. The red solid line indicates mean difference for the dyslexia group (*M* = 0.245) and the blue solid line indicates mean difference for the TD group (*M* = −0.107). Dashed lines indicate upper and lower confidence intervals.

Figure 2 shows individual differences in MLTU between the oral and written tasks in the dyslexia group (*n* = 16, mean difference = 0.245) and the TD group (*n* = 33, mean difference = −0.107). The 95% confidence intervals overlap completely, again indicating that the difference is not statistically significant.

#### 4.2.2 Subordination Index (SI)

There was no significant main effect of modality, *F*(1, 47) = 0.510, *p* = 0.479, no significant effect of group *F*(1, 47) = 1.014, *p* = 0.319 and no significant interaction between group and modality *F*(1, 47) = 0.834, *p* = 0.366. This suggests that modality did not affect the proportion of complete clauses per T‐unit of the participants in this sample.

**FIGURE 3:**
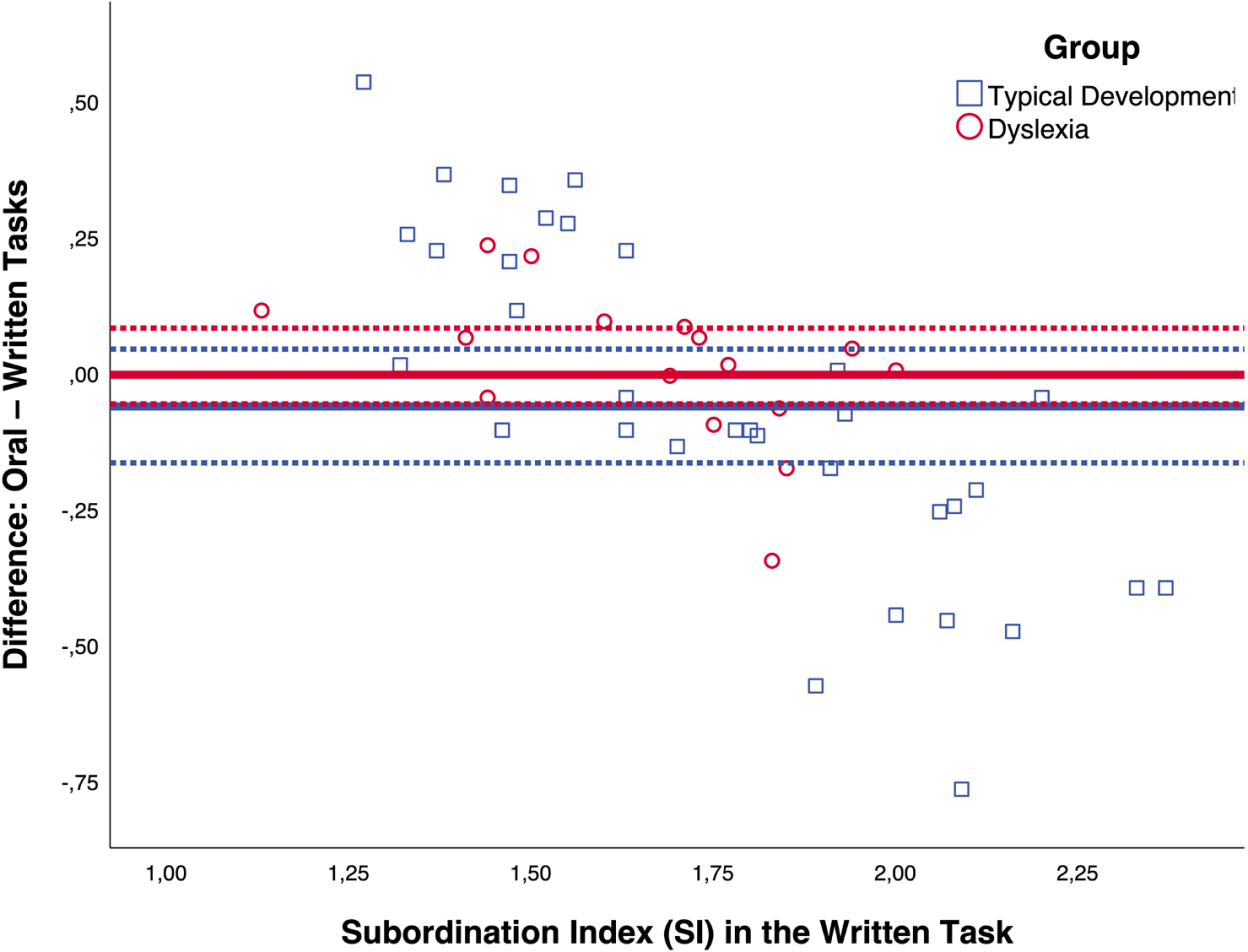
Difference between the oral and written tasks in terms of subordination Index (SI). Dots represent individual participants. The red solid line indicates mean difference for the dyslexia group (*M* = 0.01) and the blue solid line indicates mean difference for the TD group (*M* = −0.07). Dashed lines indicate upper and lower confidence intervals.

Figure 3 shows individual differences in the number of clauses per T‐unit (SI) between the oral and written tasks for the dyslexia group (*n* = 16, mean difference = 0.01) and the TD group (*n* = 33, mean difference = −0.107). The 95% confidence intervals overlap completely, again indicating that the difference is not statistically significant.

### 4.3 Linguistic Errors

#### 4.3.1 Percentage of T‐Units With Linguistic Errors

There was a main effect of modality, *F*(1, 47) = 10.798, *p* = 0.002, Λ = 0.813, ηp^2^ = 0.187, with a significantly higher proportion of T‐units with linguistic errors in the written task (*M* = 13.5, SD = 9.94) than in the oral task (*M* = 9.2, SD = 7.31). There was no significant effect of group *F*(1, 47) = 3.015, *p* = 0.089 and no significant interaction between group and modality *F*(1, 47) = 1.717, *p* = 0.196. This suggests that modality affected the linguistic accuracy of the participants in this sample, but that there were no significant differences between the two groups.

**FIGURE 4:**
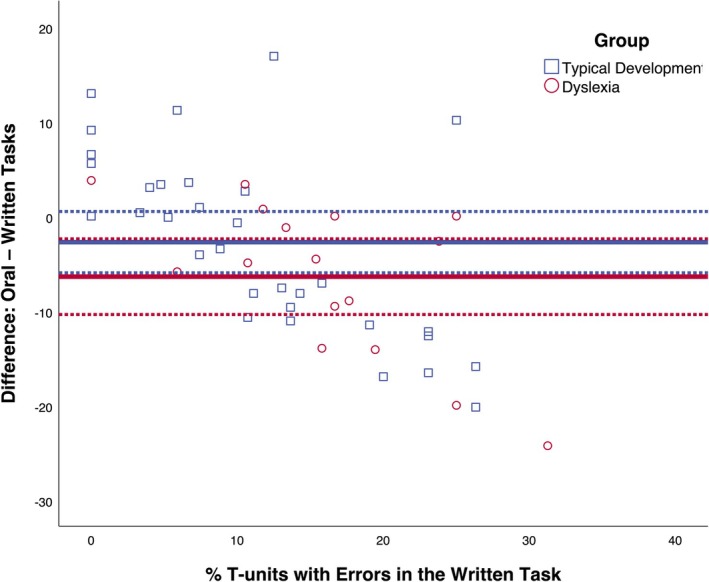
Difference between the oral and written tasks in terms of proportion of T‐units with linguistic errors. Dots represent individual participants. The red solid line indicates mean difference for the dyslexia group (*M* = 6.4%) and the blue solid line indicates mean difference for the TD group (*M* = 2.75%). Dashed lines indicate upper and lower confidence intervals.

Figure 4 shows that individual differences in the percentage of T‐units with linguistic errors between the oral and written tasks were larger in the dyslexia group (*n* = 16, mean difference = −6.40%) than in the TD group (*n* = 33, mean difference = −2.75%). However, the 95% confidence intervals overlap completely, indicating that the difference is not statistically significant.

### 4.4 Macrostructure

#### 4.4.1 Expository Scoring Scheme (ESS) Scores

There was a significant main effect of modality, *F*(1, 47) = 63.727, *p* < 0.001, Λ = 0.424, ηp^2^ = 0.576, with participants scoring higher on the ESS rubric in the oral task (*M* = 32.5, SD = 6.95) than in the written task (*M* = 26.4, SD = 6.69). There was no significant main effect of group, *F*(1, 47) = 1.603, *p* = 0.212 and no significant interaction between group and modality, *F*(1, 47) = 1.008, *p* = 0.321. This suggests that modality affected the total ESS score in this sample, but that there were no significant differences between the two groups.

**FIGURE 5:**
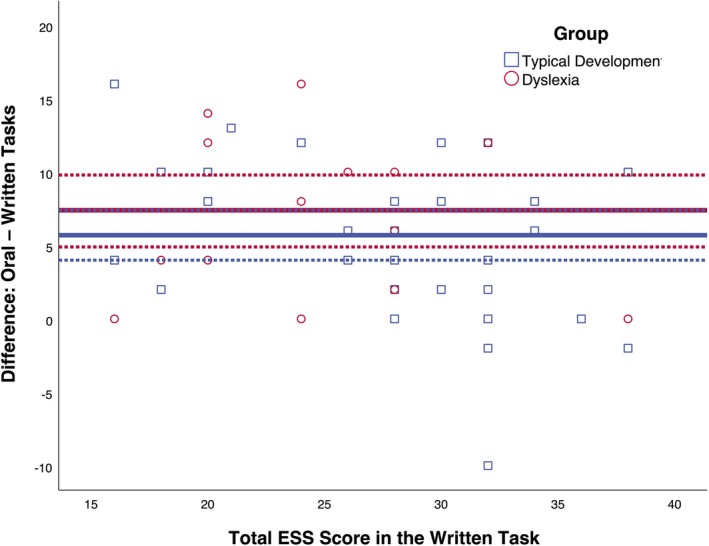
Difference between the oral and written tasks in terms of total ESS score. Dots represent individual participants. The red solid line indicates mean difference for the dyslexia group (*M* = 7.4) and the blue solid line indicates mean difference for the TD group (*M* = 5.7). Dashed lines indicate confidence intervals.

Figure 5 shows that individual differences in the total ESS score between the oral and written tasks were larger in the dyslexia group (*n* = 15, mean difference = 7.4) than in the TD group (*n* = 17, mean difference = 5.7). Again, the 95% confidence intervals overlap, indicating that the difference is not statistically significant.

### 4.5 Spelling

#### 4.5.1 Proportion of Misspelled Words

Results from independent‐samples two‐tailed *t*‐test (equal variances not assumed) showed that the dyslexia group (*M* = 8.5, SD = 5.1) had a significantly higher proportion of misspelled words than the TD group (*M* = 1.3, SD = 1.5; *t*(16.24) = 5.5, *p* < 0.001, two‐tailed). The magnitude of the differences in the means (mean difference = 0.07, 95% CI [0.044, 0.099]) was large (*d* = 2.30).

In summary, none of the measures, except from misspelled words, differed significantly between the groups. A trend was observed in the proportion of linguistic errors, however (*p* = 0.089), with more linguistic errors in the written samples of the dyslexia group on average (*M* = 16.2%) than in the TD group (*M* = 12.2%). In addition, comparison of the average group differences between the oral and written tasks showed greater differences in total number of words, ESS scores and proportion of linguistic errors in the dyslexia group than in the TD group, whereas differences in syntactic complexity (MLTU and SI) were minimal or absent.

## 5 Discussion

The aims of the present study were to compare oral and written expository skills in Swedish adolescents with and without dyslexia and investigate the effect of modality, to highlight the potential impact of transcribing and decoding difficulties on written text quality (productivity, complexity and accuracy) and potentially subtle non‐phonological language difficulties on oral and written expository discourse production. The adolescents with dyslexia were expected to show difficulties in the written task compared to peers based on theory and previous research, but this was not confirmed by the results; in fact, there were no significant effects of group on any of the measures in either task, except for proportion of misspelled words, consistent with spelling being a core difficulty in dyslexia (Berninger et al. 2008; Connelly et al. 2006). With regard to modality, we expected both groups to have higher productivity, more content and higher linguistic accuracy in the oral task compared to the written task and our results were in line with this prediction. We also expected that this modality effect would be more pronounced in the dyslexia group due to increased transcription and decoding demands in writing, but this was not supported by the data.

### 5.1 Group Effects

The non‐significant group differences in both the oral and written discourse tasks raise the possibility that non‐phonological discourse‐level difficulties reported in earlier studies may partly reflect influence of non‐reported co‐occurring conditions such as DLD or ADHD, rather than being a consequence of dyslexia per se. In the present study, groups performed similarly on standardised tests of grammatical comprehension and non‐word repetition, but there was a significant group difference on the sentence repetition task, which is a well‐known ‘marker’ for DLD. The mean difference between groups was less than one standard deviation, however and the range of scores indicates performance within the typical range also in the dyslexia group (cf. Bishop et al. 2009; Ramus et al. 2013). In addition, a DLD diagnosis was excluded for all participants through a comprehensive language assessment by a certified SLP. Previous studies differ on how language abilities have been assessed and reported and whether participants with additional diagnoses were excluded (Cabbage et al. 2026). In a recent meta‐analysis, Graham et al. (2021) reported significant writing difficulties beyond spelling in children with reading difficulties (RD) compared to peers. Their broader RD criteria, encompassing word reading and/or comprehension deficits, likely also included individuals with language difficulties or undiagnosed DLD (Adlof and Hogan 2018). Some of the studies including students with dyslexia have included assessment of language in addition to cognitive abilities (Berninger et al. 2008; Puranik et al. 2007). Others focused primarily on cognitive measures or literacy outcomes (Connelly et al. 2006; Sumner et al. 2013). These methodological differences may contribute to the variability in findings across the literature and highlight the importance of carefully described samples in future dyslexia research, both regarding linguistic skills and comorbidity, something that was recently highlighted in the systematic review by Cabbage et al. (2026).

However, the absence of significant group differences in the present study may also be explained by a relatively small sample size and large individual variability within both groups, which likely reduced the statistical power. It is possible that group differences would emerge in a larger sample, since research suggests that individuals with dyslexia may show difficulties with non‐phonological language skills (Adlof and Hogan 2018; Bishop et al. 2009; Ramus et al. 2013) and written discourse skills (Berninger et al. 2008; Connelly et al. 2006; Sumner et al. 2013; Wengelin et al. 2014). We therefore discuss a few non‐significant results that nonetheless showed mean differences between the groups, specifically: linguistic errors in the written task, number of words in the oral task and ESS scores in the written task.

The tendency towards a higher proportion of T‐units with linguistic errors in the written samples of the dyslexia group compared to the TD group was in line with the results in Puranik et al. (2007). That this pattern was only seen in the written task could indicate that this is a consequence of the increased demands of the written modality, rather than evidence of non‐phonological language difficulties (Berninger and Amtmann 2003). This suggestion is further supported by the fact that the participants with dyslexia tended to produce more words in the oral task compared to the participants with TD on average, while written productivity was comparable across groups. It is thus possible that written productivity was somewhat limited by the reading and spelling difficulties that characterise dyslexia, as suggested in previous research (Connelly et al. 2006; Sumner et al. 2013).

In the same line of reasoning, transcription skills together with limitations in working memory and other executive functions were expected to have a negative impact on content and organization in the written samples in the dyslexia group. The results showed a great overlap between ESS scores across both groups and non‐significant differences, but with a slightly lower mean ESS score in the written task of the dyslexia group, despite the comparable written sample length. Previous research has also shown mixed results (Berninger et al. 2008; Connelly et al. 2006; Herbert et al. 2018; Sumner and Connelly 2020) and comparisons are challenging since macrostructure measures are inconsistent across studies (Guilkey and Wagovich 2022) if they are at all reported (Heilmann and Malone 2014).

### 5.2 Modality Effects

Modality effects similar to the present study with respect to productivity, linguistic accuracy and overall quality have previously been reported in studies of younger school‐aged TD children (Fey et al. 2004; Scott and Windsor 2000) and our results support that these modality effects are at play into later school‐years, both in adolescents with TD and adolescents with dyslexia. That this pattern was not less pronounced in adolescents with TD was somewhat surprising, but might be a reflection of that oral and written expository tasks place high demands on both language skills and executive functioning skills, also for adolescents with TD (Nippold et al. 2005, 2008). Participants described the different topics listed on the planning sheet in more detail in the oral task compared to the written task, with longer samples and higher ESS scores as a result. As eight out of 10 ESS components correspond to topics on the planning sheet, the level of detail with which these topics are described is highly important for the total score. It is possible that a more fine‐grained analysis of the different sub‐components of the ESS would give more information on modality effects, but the total ESS score has been shown to have higher inter‐ and intra‐rater reliability than the components (Kay‐Raining Bird et al. 2016). To focus on the total ESS score is therefore our recommendation, also clinically.

The absence of modality effect for syntactic complexity was consistent with the findings of Scott and Windsor (2000) but contrasted with those of Brimo and Hall‐Mills (2019), which can be explained by differences in the measures used. Instead of using MLTU or subordination index, Brimo and Hall‐Mills (2019) used the proportion of complex utterances and found that adolescents had a higher proportion in written expository samples than in spoken expository samples.

### 5.3 Methodological Considerations

Several methodological factors may have contributed to our results. The FGS task allows participants to select a familiar and personally relevant topic and provides structural support through a planning sheet. The scaffold likely reduced the cognitive load and supported both content organization and productivity (Guilkey and Wagovich 2022). In the present study, one of the participants with dyslexia commented spontaneously after completing the writing task that he had never written such a long text before. It is possible that factors such as topic interest, background knowledge and motivation may be of particular importance to students who struggle with writing. The importance of background knowledge and motivation for expository production has also been highlighted by Nippold (2009) and Lundine and McCauley (2016). In addition, 14 out of 16 participants with dyslexia had already met the test leader on one occasion for a language, reading and writing assessment and the expository tasks were similar to other language, reading and writing tasks they had already completed with the SLP. Thus, those participants might have felt more comfortable in the test situation compared to those who completed the tasks in a research situation only.

Inter‐rater reliability varied across measures and was generally good to excellent, except for the measure of linguistic errors. Reliability was acceptable for the written samples (85%) but low in the oral samples (71%). The lower reliability in the oral context is likely attributable to greater variability in what is considered acceptable in spoken language. Previous research has shown that the proportion of T‐units with linguistic errors in spoken language samples is a measure for which it is difficult to achieve inter‐rater consensus, even when using SALT's own databases (Morley et al. 2014). Disagreements reflect both difficulties in agreeing what should be coded as an error in spoken language and the disproportionate impact of single coding differences in samples with few errors. Consequently, the generalizability of results related to linguistic errors in the oral samples is limited. However, because the same rater coded all samples from the participants with dyslexia and a majority of the samples from the participants with TD, the patterns observed remain informative. Expanding the transcription manual with additional linguistic error examples is an important direction for future work and may lead to higher inter‐rater agreement.

## 6 Conclusions

The adolescents with dyslexia included in this study performed comparably to their peers in an oral and written expository discourse task on both micro‐ and macrolevel measures, apart from proportion of misspelled words in the written task. In addition, modality affected the groups in a similar way; both the dyslexia and TD groups produced oral samples that had more words, higher linguistic accuracy and more content compared to their written samples. The results suggest that the decoding and spelling difficulties associated with dyslexia do not impair written discourse quality regarding content, productivity, complexity and linguistic accuracy in a structured and scaffolded expository task and that subtle non‐phonological language difficulties did not affect oral discourse production in this task to a significant extent in our group of adolescents with dyslexia. Thus, when a student exhibits or is reported to have writing difficulties beyond spelling, language needs to be thoroughly assessed, as such difficulties may reflect underlying language weaknesses, including DLD. To further clarify the influence of non‐phonological language skills on discourse production in adolescents with literacy challenges, future studies should explore oral and written expository skills in adolescents with dyslexia only, dyslexia with comorbid DLD and DLD only.

## Funding

This study was partly supported by the Aina Börjeson Foundation for Speech Language Pathology Research and Treatment.

## Conflicts of Interest

The authors declare no conflicts of interest.

## Data Availability

The data that support the findings of this study are available on request from the corresponding author. The data are not publicly available due to privacy or ethical restrictions.
